# P055. Prevalence of migraine in subclinical hypothyroidism: a case-control study

**DOI:** 10.1186/1129-2377-16-S1-A81

**Published:** 2015-09-28

**Authors:** Innocenzo Rainero, Elisa Rubino, Costanza Vicentini, Francesca Garino, Federico Ragazzoni, Lorenzo Pinessi, Paolo Limone

**Affiliations:** Headache Center, Department of Neuroscience “Rita Levi Montalcini”, University of Torino, Turin, Italy; Division of Endocrinology Diabetes and Metabolism, Department of Medicine, A.O. Ordine Mauriziano, Turin, Italy

## Background

The existence of an association between migraine and thyroid diseases is still a matter of debate. Epidemiological studies have shown a potential association between plasma TSH concentrations and migraine but results are contradictory. Subclinical hypothyroidism (SCH) is a common clinical entity defined biochemically as a normal serum free thyroxine (fT4) concentration in the presence of an elevated serum thyroid-stimulating hormone (TSH) concentration. In population-based studies, the prevalence of SCH ranges from 4 to 15 percent, the diagnosis being more common in women and in old age. Prospective data have shown that SCH is a risk factor for coronary heart disease, heart failure, and cardiovascular mortality, probably related to a mechanism of endothelial dysfunction and low grade chronic inflammation. A recent study suggested an increased prevalence of subclinical hypothyroidism in childhood migraine.

The purpose of this study was to evaluate the prevalence and clinical characteristics of migraine in patients with subclinical hypothyroidism.

## Methods

A case-control clinical study was conducted to investigate the prevalence of migraine in 75 consecutive patients (66 females, 9 males; mean age ± SD: 52.8 ± 14.9 yrs) with subclinical hypothyroidism and in 120 matched healthy controls. Detailed information about migraine was obtained using a structured questionnaire at a face-to-face interview. Moreover, TSH, fT4, anti-thyroid peroxidase antibody (TPO-ab), and anti-thyroglobulin (TG-ab) antibodies were measured in SCH patients.

## Results

The prevalence of lifetime migraine in SCH patients was significantly higher than that in controls (62% vs 18%; p < 0.001; odds ratio = 7.43; 95% confidence interval = 3.88-14.25). Percentage of migraine with aura versus migraine without aura (MA/MO) was significantly higher in SCH patients than controls (p = 0.03).

TSH, fT4, TPO-ab and TG-ab concentrations were not significantly different between SCH patients with migraine and without migraine.

## Conclusions

Our data suggest that patients with subclinical hypothyroidism have a higher risk of lifetime migraine than controls. Additional studies are needed in order to confirm this association and to investigate potential mechanisms of this comorbidity.

Written informed consent to publication was obtained from the patient(s).

